# Donor and corneal tissue characteristics at an eye bank in Medellín, Colombia: a cross-sectional study

**DOI:** 10.1007/s10561-026-10263-x

**Published:** 2026-11-12

**Authors:** Jhonny Alexander Castaño Morales, Juana Hoyos Palacio, Laura Nataly Higuita Duque, María Alejandra Montoya Silva, Pamela Arroyo Ibargüen, Miguel Ángel Castaño Morales, Jorge Hernando Donado Gómez, Ana Isabel Rivera Gómez

**Affiliations:** 1 Department of Ophthalmology, Universidad Pontificia Bolivariana, UPB, Medellín, Antioquia Colombia; 2 Department of Ophthalmology & Visual Sciences, Research Institute of the McGill University Health Centre, McGill University, Montreal, Quebec, Canada; 3 Universidad EIA , Escuela de Ingenieros de Antioquia, Medellín, Antioquia, Colombia; 4 Universidad Pontificia Bolivariana , UPB, Medellín, Antioquia, Colombia; 5 Research Department, Hospital Pablo Tobón Uribe, Medellín, Antioquia, Colombia

**Keywords:** Corneal transplantation, Corneal donors, Eye banks, Colombia

## Abstract

Corneal opacity remains an important cause of visual impairment worldwide, and keratoplasty is a key treatment for several corneal conditions. The persistent disparity between clinical demand and donor tissue availability underscores the importance of eye banking in ensuring appropriate tissue evaluation, preservation, and allocation. This study aimed to characterize donor corneal tissue (DCT) demographics, procurement logistics, tissue quality parameters, and surgical utilization at a regional eye bank in Medellín, Colombia, during 2025. A retrospective cross-sectional study was conducted using DCT records from an eye bank in Medellín, Colombia, between January 1 and December 31, 2025. Evaluated variables included donor sociodemographic characteristics, procurement logistics, slit-lamp biomicroscopic findings, specular microscopy parameters, and surgical utilization. A total of 274 DCT records were evaluated, of which 23 (8.4%) were discarded and not used for transplantation. Reactive serological results accounted for 18 discarded DCTs (syphilis, n = 16; human immunodeficiency virus, n = 2), while 5 were excluded for non-serological structural or tissue-quality findings (corneal foreign bodies, n = 2; numerous Descemet membrane folds, n = 2; poor intraoperative tissue quality, n = 1). Among the 256 seronegative DCTs undergoing secondary evaluation, the median donor age was 41.5 years (IQR, 26.0–55.3). Specular microscopy showed a median endothelial cell density (ECD) of 3155 cells/mm^2^ (IQR, 2994–3413), coefficient of variation (CV) of 41% (IQR, 38–44%), and hexagonality (HEX) of 48% (IQR, 45–53%). The median death-to-tissue procurement time was 8.0 h (IQR, 3.0–12.0) across all procurement modalities. Among isolated corneoscleral button procurements, the median death-to-preservation time was 9.0 h (IQR, 3.0–12.0). Of the 251 DCTs successfully transplanted, penetrating keratoplasty (PK) was the most frequent surgical approach (77.2%), followed by deep anterior lamellar keratoplasty (DALK; 12.7%), Descemet membrane endothelial keratoplasty (DMEK; 3.1%), and Descemet stripping automated endothelial keratoplasty (DSAEK; 3.1%). DCTs evaluated at a regional eye bank in Medellín were characterized by a relatively young donor population, a median endothelial cell density of 3155 cells/mm^2^, and procurement and preservation intervals within accepted timeframes. PK was the predominant surgical approach among transplanted tissues. Despite the limitations inherent to retrospective secondary data collection, these findings provide baseline evidence on donor tissue characteristics, procurement processes, and surgical utilization and may serve as a reference for future eye banking studies in Colombia and Latin America.

## Introduction

Corneal opacity represents the fifth leading cause of avoidable blindness worldwide, accounting for approximately 3.2% of global blindness cases and representing a substantial public health burden that remains largely treatable through corneal transplantation (Flaxman et al. 2017). Although keratoplasty is the most widely performed allogeneic tissue transplantation worldwide, the shortage of donor tissue remains a major challenge. Globally, an estimated 12.7 million individuals were awaiting corneal transplantation, while only approximately 185,000 procedures were performed annually in 2012 (Gain et al. 2016). Nevertheless, donor tissue procurement has increased in several regions in recent years. In Europe, donor tissue collection increased from 17,989 grafts in 2007 to 37,457 in 2024 (Kuklinski et al. 2026), while in Latin America, Brazil performed 38,728 corneal transplants over the last decade, reaching a peak of 5097 procedures in 2017 (Loureiro et al. 2026).

In Colombia, official data from the National Institute of Health (Instituto Nacional de Salud, INS) indicated that nearly 1000 patients were actively registered on the national waiting list for corneal transplantation by the end of 2022. A substantial disparity persists between tissue availability and clinical demand, as reflected by a national organ donation rate of 6.4 per million inhabitants compared with a transplantation rate of 23.2 per million (INS 2023). In this context, the operational efficiency, technical rigor, and quality assurance protocols implemented by regional eye banks are important for maximizing the utilization of available donor tissue and minimizing tissue discard (Li 2022).

Within this context, this study aimed to characterize donor corneal tissue (DCT) obtained from deceased donors and processed at an eye bank in Medellín, Colombia, between January 1 and December 31, 2025. Specifically, we evaluated procurement logistics, donor demographic and clinical characteristics, corneal parameters assessed by slit-lamp biomicroscopy and specular microscopy, and surgical utilization. By examining these operational and biological parameters, this study provides a comprehensive description of DCT procurement and eye banking practices at a regional eye bank in Medellín, Colombia.

## Methods

### Study setting and regulatory framework

The eye bank in Medellín, Colombia, is a private, non-profit institution responsible for the procurement, processing, evaluation, and distribution of ocular tissues, including corneas and sclera, intended for transplantation. Its multidisciplinary team comprises a cornea specialist, a scientific-technical coordinator, a quality assurance manager, and certified eye bank technicians. The eye bank is regulated by the Colombian National Donation and Transplantation Network under the oversight of the National Institute of Health (Instituto Nacional de Salud, INS), in accordance with Decree 2493/2004, Resolution 5108/2005, and Law 1805/2016.

### DCT procurement and preservation protocol

Current national guidelines establish a maximum death-to-procurement interval of 15 h for non-refrigerated donors. Because no fixed time limit is specified for refrigerated bodies, procurement is initiated promptly upon notification by on-call technical staff. Upon donor identification, the treating healthcare facility notifies the eye bank, which dispatches certified technicians to the hospital or the National Institute of Legal Medicine and Forensic Sciences (Instituto Nacional de Medicina Legal y Ciencias Forenses) for tissue procurement. The procurement is performed according to standardized aseptic surgical protocols by technicians trained by the eye bank.

Ocular tissues may be procured using one of two methods: as isolated corneoscleral buttons or as whole globes en bloc.

For isolated corneoscleral buttons, the tissue was directly immersed in Eusol-C® storage medium at the procurement site and maintained in continuous immersion until surgical use to prevent contamination. Conversely, when whole globes were excised en bloc, each globe was placed in a sterile container over sterile gauze, ensuring no direct contact between the globe and the container. Three drops of 0.3% gentamicin were applied topically to the cornea, and the container was filled to one-third of its capacity with normal saline solution.

All harvested tissues, regardless of whether they were isolated corneoscleral buttons or whole globes, were transported to the eye bank facility in insulated coolers packed with wet ice to maintain a temperature of 0–10 °C. Upon arrival at the eye bank, the processing workflow differed according to the procurement method. Isolated corneoscleral buttons were placed directly into refrigeration at 0–10 °C, whereas for whole globes, the corneoscleral button was subsequently excised under sterile conditions within a Class II biosafety cabinet equipped with a high-efficiency particulate air (HEPA) filtration system. The button was then immediately immersed in Eusol-C® preservation medium and stored at 0–10 °C in a sealed vial until surgical use.

To prevent contamination, the corneoscleral button was not removed from the preservation medium or transferred to a different vial after its initial placement. It remained sealed in the original vial until opened by the surgeon for transplantation.

### DCT suitability

All steps of the DCT evaluation process were conducted by certified eye bank personnel using a two-step approach. First, mandatory primary serological screening was performed on serum samples from all corneal donors to assess for transmissible infectious diseases. Testing began with rapid screening assays, followed by confirmatory serum testing for syphilis, human immunodeficiency virus (HIV), hepatitis B, and hepatitis C. Second, tissues that passed the serological screening underwent secondary structural and endothelial evaluation using two standardized assessment modalities to determine their suitability for surgical allocation.

The first modality, slit-lamp biomicroscopy, assessed corneal structural integrity across all layers, including epithelial and stromal defects, edema, opacities and foreign bodies; Descemet membrane folds; specular reflex; and peripheral corneal alterations. The second modality, semi-automated specular microscopy, evaluated endothelial cell parameters using the Konan CellChek D + Specular Microscope, specifically recording endothelial cell density (ECD), mean cell area (MCA), pachymetry, polymegathism quantified as the coefficient of variation in cell area (CV), and hexagonality (HEX). Structural endothelial abnormalities, including guttae and endothelial excavations, were also systematically documented.

For each cornea, two distinct fields were captured and analyzed. In each field, an initial automated algorithmic trace was generated, followed by operator validation and manual correction to ensure accurate delineation of cell boundaries, with a minimum of 75 cells evaluated per field. Final measurements were automatically averaged by the software across all analyzed fields to obtain the definitive values for ECD, MCA, pachymetry, CV, and HEX.

### DCT disqualification criteria

DCTs were considered unsuitable for therapeutic transplantation, whether refractive or tectonic, and were discarded if any of the following conditions were present: a reactive or confirmed positive result on donor infectious disease serology screening; the presence of any stromal or endothelial foreign body; or severe Descemet membrane folds classified as numerous or very numerous on slit-lamp biomicroscopic examination.

### DCT allocation criteria

DCTs were classified by the eye bank as suitable for either refractive or tectonic keratoplasty based on predefined microscopic and structural parameters. Refractive keratoplasty was reserved for high-quality grafts meeting strict endothelial and structural criteria, including an endothelial cell density (ECD) ≥ 2400 cells/mm^2^, coefficient of variation (CV) ≤ 75%, hexagonality (HEX) ≥ 51%, epithelial defects affecting < 50% of the corneal surface, stromal edema involving < 50% of the corneal surface, and absence of central opacities.

In contrast, DCTs with moderate endothelial or structural compromise were allocated for tectonic keratoplasty. These grafts met one or more of the following criteria: ECD < 2400 cells/mm^2^, CV > 75%, HEX < 51%, epithelial defects affecting ≥ 50% of the corneal surface, stromal edema involving ≥  50% of the corneal surface, presence of central opacities, mild-to-moderate Descemet membrane folds, presence of guttae or endothelial excavations, or an arcus senilis measuring > 3 mm.

### DCT storage and transport

The maximum allowable storage period was 14 days for refractive corneas maintained in Eusol-C® preservation medium under refrigeration at 0–10 °C. Tectonic corneas were also maintained in Eusol-C® preservation medium but were frozen at temperatures ranging from − 1 °C to − 65 °C for up to 6 months. DCTs that were not allocated or deemed suitable for transplantation within the designated storage periods were considered unsuitable, discarded, and incinerated in accordance with standard biosecurity protocols.

During final distribution, DCTs from both groups were transported in specialized coolers containing wet ice and maintained between 0 and 10 °C, regardless of their intended use for refractive or tectonic keratoplasty. Each shipment was accompanied by official documentation detailing the unique graft identification number, endothelial cell parameters, and negative donor serology results, in accordance with applicable Colombian regulations.

### Study design and data collection

All DCT records received between January 1, 2025, and December 31, 2025, were evaluated. A complete census of all DCT records available during the study period was conducted. Data were retrieved from secondary institutional eye bank records and systematically compiled into a database developed by the investigators. Data extraction and database coding were performed by a single investigator without independent double verification; therefore, potential transcription errors could not be completely excluded.

Statistical analyses were performed using SPSS® software. Quantitative variables were summarized using measures of central tendency and dispersion according to their distribution, which was assessed using the Shapiro–Wilk test. Qualitative variables were summarized using absolute and relative frequencies.

### Ethical considerations

The study was conducted in accordance with the ethical principles for medical research involving human subjects outlined in the Declaration of Helsinki and applicable national regulations. The study protocol was approved by the Research Ethics Committee of Pontificia Universidad Bolivariana. The study was classified as minimal-risk research; therefore, the requirement for individual informed consent was waived.

### Variable definitions

To ensure consistency and reproducibility, study variables were operationally defined according to the assessment modality and stage of the DCT workflow.

#### Variables assessed by slit-lamp biomicroscopy

- *Epithelial defect:* defined as focal loss of epithelial tissue, surface excavation, or detached epithelial flaps. Severity was graded according to the percentage of total corneal surface involved: 0 (absent), 1 ( < 25%), 2 (25–50%), 3 (51–75%), and 4 ( >  75%).
- *Epithelial edema:* defined by superficial epithelial thickening with an ill-defined, grayish, or cloudy appearance. Severity was graded according to the percentage of total corneal surface involved: 0 (absent), 1 ( <  25%), 2 (25–50%), 3 (51–75%), and 4 ( > 75%).
- *Stromal opacities:* defined as hazy or dense opaque areas within the stromal matrix. Findings were categorized according to anatomical location as absent, central, or peripheral.
- *Epithelial opacities:* defined as hazy or dense opaque areas within the epithelial layer. Findings were categorized according to anatomical location as absent, central, or peripheral.
- *Descemet membrane folds:* defined as linear striations or folds involving Descemet membrane. Their extent was classified using a six-category scale: absent, few, mild, moderate, numerous, or very numerous.
- *Peripheral corneal changes:* defined as structural alterations located outside the central optical zone. Findings were categorized as absent, arcus senilis, pterygium, or other. Arcus senilis was defined as a concentric peripheral corneal lipid deposition separated from the limbus by a clear zone. Pterygium was defined as triangular fibrovascular tissue extending across the limbus. Other findings included marginal scars, peripheral thinning, and postsurgical changes.
- *Specular reflex:* defined as the appearance and uniformity of reflected light from the corneal tissue–medium interface. Findings were classified as small, indicating a smooth and intact endothelial surface, or large, indicating disruption of the endothelial surface.
- *Epithelial foreign bodies:* defined as foreign material or particulate matter embedded within or adherent to the corneal epithelium and recorded as present or absent.
- *Stromal foreign bodies:* defined as foreign material or particulate matter embedded within or adherent to the stromal matrix and recorded as present or absent.
- *Stromal edema:* defined as loss of stromal clarity, corneal thickening, and diffuse stromal haze and recorded as present or absent.

### Variables assessed by specular microscopy

#### Quantitative variables

- *Endothelial cell density (ECD, cells/mm*^*2*^*):* number of endothelial cells per square millimeter of posterior corneal surface.
- *Mean cell area (MCA, µm*^*2*^*):* average area of individual corneal endothelial cells.
- *Coefficient of variation (CV, %):* coefficient of variation of individual endothelial cell areas, reflecting the degree of variation in cell size.
- *Hexagonality (HEX, %):* percentage of endothelial cells with a six-sided configuration among all analyzed cells.
- *Pachymetry (µm):* measurement of central corneal thickness.

#### Qualitative variables

- *Endothelial folds:* defined as linear striations or folds observed on the posterior surface of the cornea, recorded as absent or present.
- *Endothelial guttae:* defined as the presence of small protrusions or dark spots on the posterior corneal surface, recorded as absent or present.

#### Procedural and temporal variables

- *Procurement complications:* adverse events occurring during tissue procurement that compromised graft structural integrity, including lacerations, irregular cuts, or epithelial defects.
- *Death-to-tissue-procurement time (hours):* interval between the officially recorded time of donor death and tissue procurement, defined as either en bloc globe enucleation or isolated corneoscleral button procurement.
- *Death-to-preservation time (hours):* total elapsed time between the recorded time of donor death and placement of the isolated corneoscleral button into preservation medium. This interval was not quantifiable for corneas procured through whole-globe enucleation because the corresponding time was not routinely recorded in the institutional database.
- *Storage duration (days):* elapsed time between initial tissue procurement and final release of the tissue for surgical use.
- *Surgeon-rated tissue quality:* qualitative assessment of DCT performance reported by the operating surgeon based on macroscopic appearance, intraoperative handling, and overall suitability for the intended surgical procedure. Tissue quality was categorized as excellent, very good, good, fair, or poor.

## Results

**Fig. 1:**
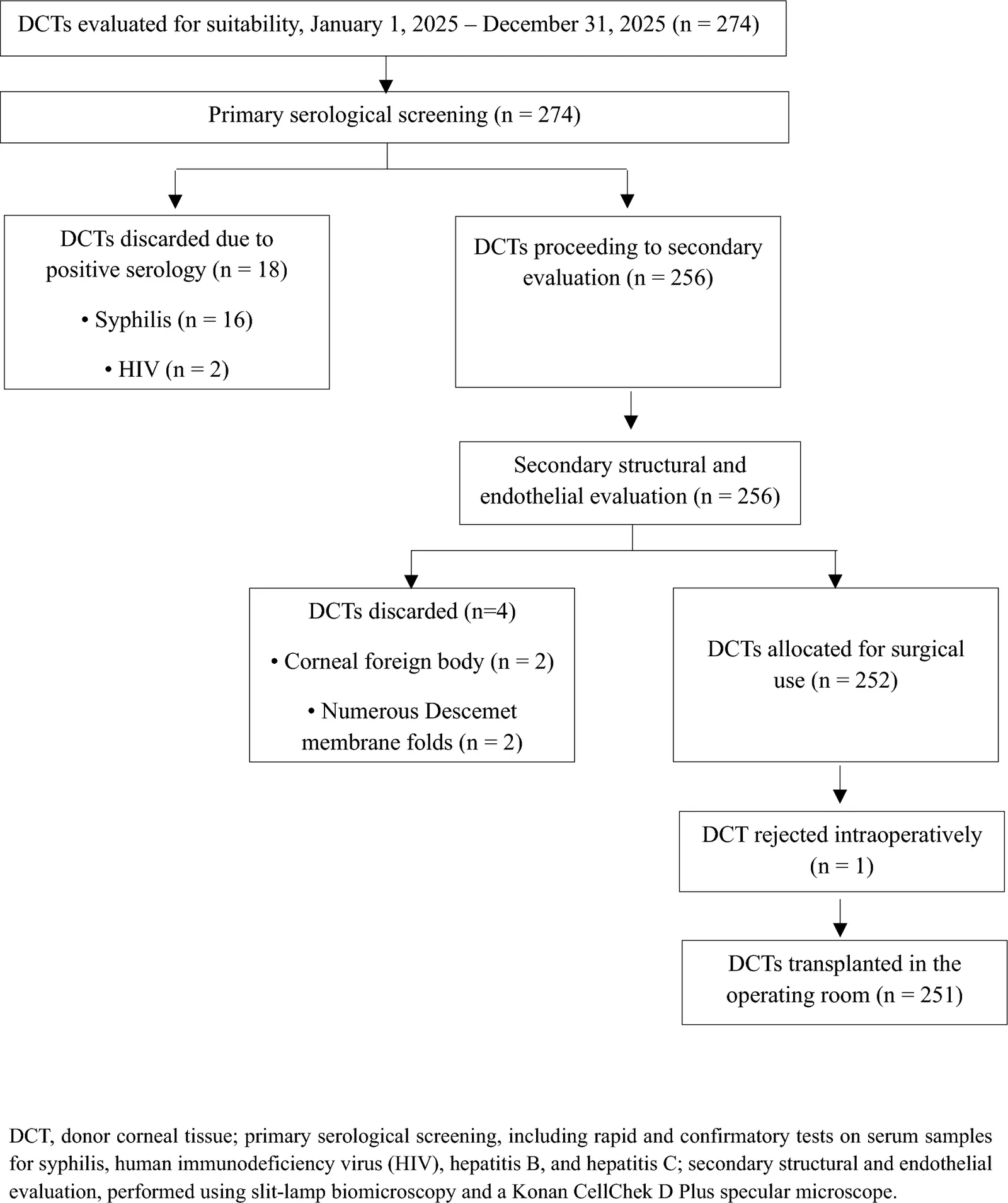
Flowchart of donor corneal tissue suitability, exclusion, and final clinical disposition

Of the 274 DCTs evaluated at the eye bank between January 1 and December 31, 2025, 23 (8.4%) were ultimately discarded and not used for transplantation **(**Fig. 1**)**. Serological screening showed non-reactive results in 256 of 274 DCTs (93.4%), while 18 (6.6%) had reactive or positive serological results, including syphilis (n = 16) and HIV (n = 2); no positive results were identified for hepatitis B or C. Following the exclusion of these 18 seropositive DCTs according to safety protocols, the remaining 256 seronegative DCTs proceeded to secondary structural and endothelial evaluation. During this secondary evaluation and subsequent clinical assessment, five additional DCTs were disqualified for non-serological reasons, including corneal foreign bodies (n = 2), numerous Descemet membrane folds (n = 2), and poor intraoperative tissue quality (n = 1).

The following sections present the findings of the secondary evaluation of the 256 seronegative DCTs.

### Sociodemographic and donor characteristics

The median donor age was 41.5 years (Interquartile range [IQR],  26.0–55.3 years). Male donors predominated (185/256; 72.3%), compared with female donors (71/256; 27.7%). Regarding ethnoracial background, most donors were classified as mixed ethnicity (252/256; 98.4%), followed by equal proportions of Black (2/256; 0.8%) and White (2/256; 0.8%) donors.

Chestnut iris pigmentation was predominant (218/256; 85.2%), followed by brown (22/256; 8.6%), green (10/256; 3.9%), and hazel (2/256; 0.8%). No blue-eyed donors were recorded (0/256; 0.0%), and iris pigmentation data were missing for 4/256 donors (1.6%).

Trauma was the leading cause of death (153/256; 59.8%), followed by cardiovascular (65/256; 25.4%), cerebrovascular (22/256; 8.6%), and respiratory causes (10/256; 3.9%). Other causes accounted for 2/256 cases (0.8%). No deaths due to infectious causes were recorded (0/256; 0.0%), while the cause of death was unreported in 4/256 cases (1.6%).

Most deaths occurred in hospital settings (149/256; 58.2%), followed by public thoroughfares (82/256; 32.0%) and home settings (25/256; 9.8%).

### Procurement modality, death-to-tissue-procurement time and death-to-preservation time

Isolated corneoscleral buttons accounted for 118/256 right corneas (46.1%) and 117/256 left corneas (45.7%), whereas whole globes obtained by enucleation accounted for 11/256 right eyes (4.3%) and 10/256 left eyes (3.9%). No procurement complications were reported during any tissue procurement procedure (256/256; 100%).

Across all procurement modalities (n = 256), the median death-to-tissue procurement time was 8.0 h (IQR, 3.0–12.0 h). Among isolated corneoscleral button procurements (n  =  235), for which preservation was performed immediately after procurement, the median death-to-preservation time was 9.0 h (IQR, 3.0–12.0 h). In contrast, for corneas obtained through whole-globe enucleation (n = 21), the exact interval between donor death and preservation in culture medium could not be determined because this interval is not routinely recorded in the institutional database.

### Slit-lamp biomicroscopy and specular microscopy parameters

Regarding the secondary evaluation of DCTs, slit-lamp biomicroscopy findings are summarized in Table 1, while specular microscopy findings are presented in Table 2.

**Table 1:** Slit-lamp biomicroscopy parameters of donor corneal tissues (DCTs), n = 256

Variable	Category	n (%)
Epithelial foreign bodies	Absent	254 (99.2)
	Present	2 (0.8)
	Missing data	0 (0)
Peripheral corneal changes	Absent	198 (77.3)
	Arcus senilis	45 (17.6)
	Pterygium	12 (4.7)
	Other	1 (0.4)
	Missing data	0 (0)
Epithelial defect	Absent	174 (68)
	< 25%	75 (29.3)
	25–50%	5 (2)
	51–75%	2 (0.8)
	> 75%	0 (0)
	Missing data	0 (0)
Epithelial opacities	Absent	254 (99.2)
	Central	0 (0)
	Peripheral	2 (0.8)
	Missing data	0 (0)
Epithelial edema	Absent	164 (64.1)
	< 25%	92 (35.9)
	25–50%	0 (0)
	51–75%	0 (0)
	> 75%	0 (0)
	Missing data	0 (0)
Descemet membrane folds	Absent	41 (16)
	Few	94 (36.7)
	Mild	107 (41.8)
	Moderate	12 (4.7)
	Numerous	2 (0.8)
	Very numerous	0 (0)
	Missing data	0 (0)
Specular reflex	Small	256 (100)
	Large	0 (0)
	Missing data	0 (0)
Stromal edema	Absent	42 (16.4)
	Present	213 (83.2)
	Missing data	1 (0.4)
Stromal opacities	Absent	250 (97.7)
	Central	5 (2)
	Peripheral	0 (0)
	Missing data	1 (0.4)
Stromal foreign bodies	Absent	255 (99.6)
	Present	0 (0)
	Missing data	1 (0.4)

For specific variables, missing data corresponded to a DCT that did not undergo further evaluation because an epithelial foreign body was identified and the tissue was subsequently discarded

**Table 2:** Specular microscopy parameters of donor corneal tissues (n = 256)

Parameter	Median (IQR) or n (%)
*Quantitative*	
Endothelial cell density, cells/mm^2^	3155 (2,994–3,413)
Mean cell area, µm^2^	318 (294–333)
Coefficient of variation, %	41 (38–44)
Hexagonality, %	48 (45–53)
Pachymetry µm	552 (541–566)
*Qualitative*	
Endothelial folds	
Absent Present Missing data	159 (62.1) 96 (37.h5) 1 (0.4)
Endothelial guttata	
Absent Present Missing data	202 (78.9) 53 (20.7) 1 (0.4)

Values are presented as median (interquartile range, IQR) or frequency, n (%). Missing data corresponded to one DCT that did not undergo further evaluation because an epithelial foreign body was identified and the tissue was subsequently discarded

### Geographic distribution, storage duration, and surgical utilization

Of the 252 DCTs validated and assigned for surgical use, distribution included the city of Medellín (150/252, 59.5%), other cities (58/252, 23.0%), Envigado (43/252, 17.0%), and Bello (0/252, 0%), while 1 case lacked a record of the destination city (1/252, 0.39%). The median storage duration (n = 252) was 6 days (IQR: 4–8).

Regarding surgeon-rated tissue quality, as evaluated by the surgeons, findings were as follows: very good (91/252, 36.1%), excellent (75/252, 29.7%), good (39/252, 15.4%), fair (4/252, 1.5%), and poor (1/252, 0.3%). The latter DCT was discarded without being implanted. In 42/252 cases (16.6%), the surgeon’s evaluation was not recorded.

Finally, among the 251 tissues that were effectively transplanted, the surgical approaches implemented corresponded to penetrating keratoplasty (PK; 194/251; 77.2%), deep anterior lamellar keratoplasty (DALK; 32/251; 12.7%), Descemet membrane endothelial keratoplasty (DMEK; 8/251; 3.1%), Descemet stripping automated endothelial keratoplasty (DSAEK; 8/251; 3.1%), other procedures (5/251; 1.9%), and missing data (4/251; 1.5%).

## Discussion

Among the 274 DCTs evaluated at the regional eye bank in Medellín, Colombia, the leading cause of discard was reactive serological testing, followed by the presence of corneal foreign bodies. Among the 256 seronegative DCTs that proceeded to secondary evaluation, we observed a relatively young donor population, high ECD, and death-to-preservation intervals within internationally recommended timeframes. Trauma was the leading cause of death, accounting for 59.8% of donors, which may partly explain the younger age distribution observed in this cohort. Together, these findings provide an initial characterization of donor demographics, procurement processes, and tissue quality parameters in a Colombian eye bank and establish a reference point for future evaluations of DCT procurement and utilization.

The median donor age in our cohort was 41.5 years (IQR: 26–55.2), consistent with previously reported data from Medellín (36.95 ± 14 years; Cadavid Usuga et al. 2024) and with national data (median: 27.5 years; Mora et al. 2021). Trauma was the leading cause of death in our cohort, accounting for 59.8% of donors (153/256), followed by cardiovascular causes (25.4%). The predominance of traumatic deaths may partly explain the relatively young donor profile observed in our population, given the younger age distribution typically associated with trauma-related mortality. In contrast, older donor populations have been reported in Brazil (median age: 53 years; Pessoa et al. 2019) and Europe (mean age: 55.5–65 years; Dunker et al. 2021), where differences in population demographics and mortality patterns may contribute to the older age distribution. These differences highlight the importance of considering the demographic and epidemiological context when comparing donor populations across eye banks.

Donor age is an important determinant of corneal endothelial characteristics, with younger donors generally presenting higher ECD (Shehab et al. 2022). Consistent with this, our cohort had a median ECD of 3155 cells/mm^2^ (IQR: 2994–3413), similar to the mean ECD of 3109 cells/mm^2^ reported in another Colombian cohort with a relatively young donor population (Mora et al. 2021). In comparison, lower ECD values have been reported in cohorts with older donors, including Denmark (median ECD: 2453 cells/mm^2^; mean age: 71 years; Shehab et al. 2022), Spain (mean ECD: 2701.3 ± 324.6 cells/mm^2^; mean age: 60.2 years; Zurutuza-Veillet et al. 2025), India (mean ECD: 2717 cells/mm^2^; mean age: 58 years; Munir and Munir 2022), and Mexico (mean ECD: 2884 cells/mm^2^; Macriz-Romero et al. 2026). These cross-regional differences are consistent with the known influence of donor age on ECD and may partly explain the relatively high ECD observed in our cohort. However, ECD should not be interpreted in isolation as a measure of overall tissue quality, since endothelial morphology and slit-lamp findings also contribute to donor tissue suitability.

The median death-to-preservation interval for cases involving isolated corneoscleral button procurement (n = 235) was 9 h (IQR, 3–12 h). This interval was shorter than that reported in the national cohort by Mora et al. (mean, 16.7 h) and comparable to intervals reported in Brazil (mean, 6.5 h, Pereira Cruz et al. 2021; mean, 7.25 h, Cruz et al. 2018; mean, 10.2 ± 5.5 h, Pessoa et al. 2019). Internationally, the observed interval was longer than that reported in Nepal (mean, 5.79 h; Bajracharya et al. 2020) but shorter than that reported in Denmark (median, 23.9 h; Shehab et al. 2022). Importantly, the median interval observed in our cohort remained within the 24 h maximum recommended by the European Directorate for the Quality of Medicines & HealthCare (EDQM, 2022) and the 15 h maximum established by national guidelines. From an eye-bank operational perspective, these findings indicate that tissue preservation was generally achieved within accepted timeframes in this setting. Nevertheless, the variability in death-to-preservation time suggests that logistical factors related to donor identification and tissue procurement may influence the time to preservation and may vary across study settings.

Endothelial morphology provides additional information regarding the characteristics of donor tissue. In our cohort, the median CV was 41% (IQR: 38–44%), while the median percentage of HEX was 48% (IQR: 45–53%). The CV was similar to that reported in Cuba (40.32%; Castillo et al. 2020) and lower than that reported in Mexico (58.48%; León-Domínguez et al. 2020). HEX in our cohort was intermediate compared with these populations, with reported values of 51.2% in Cuba and 42.68% in Mexico. Compared with the international cohort reported by Sinha et al. (2021), our CV was higher (41% vs. 35.9%), whereas HEX was lower (48% vs. 53.8%). These differences should be interpreted cautiously because endothelial morphology measurements may be influenced not only by donor characteristics but also by image acquisition, cell-selection criteria, instrumentation, and analytical methodology (Bruinsma et al. 2013). Accordingly, direct comparisons of CV and HEX between eye banks may be limited when assessment protocols and imaging platforms differ.

Regarding tissue utilization, PK was the most frequently performed procedure in our cohort, followed by DALK, DMEK, and DSAEK. This distribution is consistent with previously reported surgical patterns in Colombia, where PK has remained widely used compared with lamellar approaches. National data have reported corneal ectasias, particularly keratoconus and post-refractive ectasia, followed by bullous keratopathy, graft failure, and cicatricial leukomas among the main indications for transplantation (Barraquer et al. 2019). Similarly, Galvis et al. (2019) identified bullous keratopathy as the primary indication, followed by infectious keratitis, corneal endothelial dystrophies, and keratoconus. Although the present study did not systematically evaluate the underlying indication for each transplantation, the predominance of PK provides context for the clinical utilization of DCT in this setting and may reflect the continued need for full-thickness transplantation in our population.

Surgeon feedback represents a final acceptance step within the transplantation workflow rather than an additional assessment of donor-tissue quality. Formal tissue evaluation and release remain the responsibility of the eye bank and are based on standardized protocols, including serological testing, slit-lamp examination, and specular microscopy. Nevertheless, the surgeon may identify unexpected concerns during the final inspection or handling of the tissue and decide not to proceed with transplantation. In our cohort, one DCT was considered unsuitable for implantation following the surgeon's clinical inspection and was subsequently rejected. Although this represents a single observation, it highlights the importance of communicating such findings to the eye bank as part of the feedback process. Thus, surgeon feedback should be understood as a final acceptance decision that may inform eye-bank quality assurance, rather than as a separate or complementary validation of tissue quality. .

This study has several limitations. First, data collection relied exclusively on secondary sources, which may have introduced information bias due to missing records or pre-existing data-entry errors. Second, data extraction and database coding were performed by a single investigator without double data entry or independent verification by a second reviewer; therefore, transcription errors and single-observer bias cannot be entirely excluded. Third, the death-to-preservation interval could not be determined for enucleated whole globes, limiting a comprehensive assessment of this parameter across all procurement modalities. However, once whole-globe enucleation was completed, the tissue was immediately transported to the eye bank for corneoscleral button excision. Fourth, because this study was conducted at a single regional eye bank, the findings may not be generalizable to other eye banks in Colombia or Latin America, where donor characteristics, procurement logistics, and tissue-processing protocols may differ. Comparisons with previously published cohorts should also be interpreted cautiously because of differences in donor selection, preservation methods, processing instruments, imaging platforms, and reporting conventions. Finally, long-term clinical outcomes following transplantation were not evaluated, as assessing postoperative graft outcomes was beyond the scope of the present study, which focused on the characterization of donor and tissue parameters at the eye-bank level. Therefore, the relationship between the donor and tissue characteristics observed in this cohort and long-term postoperative graft performance could not be assessed.

Taken together, our findings provide a baseline description of donor characteristics, causes of death, procurement intervals, DCT parameters, and surgical utilization within a regional Colombian eye bank. The relatively young donor population, predominance of traumatic mortality, high ECD, preservation intervals within accepted timeframes, and predominance of penetrating keratoplasty (PK) as the most common surgical procedure characterize the donor, procurement, and surgical utilization profile of this setting. These findings may serve as a reference for future multicenter studies and longitudinal monitoring of eye-bank performance, particularly in evaluating factors associated with DCT quality and surgical utilization.

## Conclusion

This cross-sectional study provides a comprehensive description of DCT characteristics, slit-lamp biomicroscopy and specular microscopy parameters, tissue procurement timelines, and surgical utilization at a regional eye bank in Medellín, Colombia. The relatively young donor population, predominance of traumatic deaths, median ECD of 3155 cells/mm^2^, preservation intervals within accepted timeframes, and predominance of PK as the surgical procedure collectively characterize this population. Despite the limitations inherent to secondary data collection, these findings provide valuable baseline evidence on eye-banking practices in Medellín, Colombia,  and may serve as an initial benchmark for regional tissue procurement, processing, and utilization. Future prospective multicenter studies may build on these findings by incorporating standardized tissue assessment protocols and post-transplantation clinical outcomes to further investigate the relationship between baseline eye-bank parameters and long-term corneal graft outcomes.

## Data Availability

The data collected and analyzed during the current study are not publicly available due to institutional and ethical restrictions of the Eye Bank in Medellín, Colombia. The study was approved by the ethics committees of both the Eye Bank and the Universidad Pontificia Bolivariana. All data were handled under strict confidentiality conditions and were fully anonymized, with no personal identifiers included. In case any specific data are requested, interested researchers should contact the corresponding author with a justified request. Such requests will be evaluated by the respective ethics committees for approval. Due to the sensitive nature of the data, they cannot be made publicly available.
